# Netrin-1 in Atherosclerosis: Relationship between Human Macrophage Intracellular Levels and In Vivo Plaque Morphology

**DOI:** 10.3390/biomedicines9020168

**Published:** 2021-02-08

**Authors:** Susanna Fiorelli, Nicola Cosentino, Benedetta Porro, Franco Fabbiocchi, Giampaolo Niccoli, Francesco Fracassi, Nicolò Capra, Simone Barbieri, Filippo Crea, Giancarlo Marenzi, Viviana Cavalca, Elena Tremoli, Sonia Eligini

**Affiliations:** 1 Centro Cardiologico Monzino I.R.C.C.S., 20138 Milan, Italy; susanna.fiorelli@cardiologicomonzino.it (S.F.); nicola.cosentino@cardiologicomonzino.it (N.C.); franco.fabbiocchi@cardiologicomonzino.it (F.F.); nicolo.capra@cardiologicomonzino.it (N.C.); barbieri.simo@gmail.com (S.B.); giancarlo.marenzi@cardiologicomonzino.it (G.M.); viviana.cavalca@cardiologicomonzino.it (V.C.); elena.tremoli@cardiologicomonzino.it (E.T.); sonia.eligini@cardiologicomonzino.it (S.E.); 2 Dipartimento di Scienze Cardiovascolari e Toraciche, Fondazione Policlinico Gemelli I.R.C.C.S., Università Cattolica del Sacro Cuore, 00168 Rome, Italy; gniccoli73@hotmail.it (G.N.); francesco.fracassi@yahoo.it (F.F.); filippo.crea@policlinicogemelli.it (F.C.)

**Keywords:** monocyte-derived macrophages, Netrin-1, coronary artery plaque, atherosclerosis

## Abstract

Netrin-1 is a laminin-like protein that plays a pivotal role in cell migration and, according to the site of its release, exerts both pro and anti-atherosclerotic functions. Macrophages, key cells in atherosclerosis, are heterogeneous in morphology and function and different subpopulations may support plaque progression, stabilization, and/or regression. Netrin-1 was evaluated in plasma and, together with its receptor UNC5b, in both spindle and round monocyte-derived macrophages (MDMs) morphotypes from coronary artery disease (CAD) patients and control subjects. In CAD patients, plaque features were detected in vivo by optical coherence tomography. CAD patients had lower plasma Netrin-1 levels and a higher MDMs expression of both protein and its receptor compared to controls. Specifically, a progressive increase in Netrin-1 and UNC5b was evidenced going from controls to stable angina (SA) and acute myocardial infarction (AMI) patients. Of note, spindle MDMs of AMI showed a marked increase of both Netrin-1 and its receptor compared to spindle MDMs of controls. UNC5b expression is always higher in spindle compared to round MDMs, regardless of the subgroup. Finally, CAD patients with higher intracellular Netrin-1 levels showed greater intraplaque macrophage accumulation in vivo. Our findings support the role of Netrin-1 and UNC5b in the atherosclerotic process.

## 1. Introduction

The accumulation of macrophages into the artery wall is well recognized as a crucial process in the chronic inflammation underlying atherosclerosis, but the mechanisms involved in this process are not fully known. Macrophages are motile cells, however, and unlike other tissues, macrophages that accumulate in atherosclerotic plaque show a reduced capacity to migrate resulting in an imbalance between monocyte entry and macrophage exit [1]. The migration to and from atherosclerotic plaque is regulated by several chemoattractant mediators, and among them, the role of the neuroimmune guidance cue Netrin-1 is recently evidenced in the regulation of leukocyte trafficking [2]. Netrin-1 is a laminin-like protein mainly involved in the modulation of cell migration during development [3]. More recently, its role in pathological conditions including atherosclerosis has been evidenced, where it may play protective or deleterious functions. Thus, while Netrin-1 secreted by the endothelium in the circulation explicates cardiovascular protection reducing monocyte adhesion and migration, Netrin-1 produced by macrophages within the atherosclerotic plaque contributes to atherosclerosis progression preventing macrophage egression [4,5,6]. Several effects induced by Netrin-1 are mediated by the binding to the uncoordinated (UNC)5 family receptors and, in particular, the UNC5 homolog b (UNC5b) receptor modulates the chemorepulsive effect in the nervous system [7]. Netrin-1 and UNC5b have been detected in mouse and human atherosclerotic plaques, mainly in macrophage foam cells, where they are induced by hypoxia and oxidized low-density lipoprotein (ox-LDL) accumulation [4,8,9]. Moreover, high levels of extracellular Netrin-1 have been evidenced in macrophage-rich regions of the plaque [6]. Here, through an autocrine and/or paracrine mechanism, the interaction between Netrin-1 and UNC5b sustains the progression of atherosclerotic plaque inhibiting the monocyte and macrophage migration and favoring cell retention [6].

To study plaque macrophage characteristics, the human macrophages derived from an in vitro spontaneous differentiation of monocytes (MDMs) are used as a surrogate model commonly accepted by the scientific community. We have previously evidenced that MDMs obtained from coronary artery disease (CAD) patients are heterogeneous in morphology, with round and spindle cells co-existing in the same cell culture, and in function. Moreover, we showed that CAD patients showed a prevalence of round MDMs with a peculiar biochemical profile that is associated with the presence of rupture-prone coronary plaques detected in vivo by optical coherence tomography (OCT), a high-resolution imaging technology [10].

Little is known about the relationship between circulating Netrin-1 levels and CAD and between the intracellular Netrin-1 expression in human macrophages and plaque morphology. In this study, we will investigate the expression of Netrin-1 and its cognate receptor UNC5b in both spindle and round MDMs obtained from control subjects and CAD patients. In addition, the cellular profile delineated in patients will be related to the in vivo features of coronary plaque assessed by OCT.

## 2. Materials and Methods

### 2.1. Study Population

A total of 42 CAD patients who underwent coronary angiography due to stable angina (SA) or acute myocardial infarction (AMI), as their first ischemic heart disease event, and who showed obstructive atherosclerosis (>50% diameter stenosis by visual estimate) were enrolled at Centro Cardiologico Monzino IRCCS (Istituto di Ricovero e Cura a Carattere Scientifico). A total of 21 subjects, without history or current symptoms of coronary heart, and not taking any cardiovascular therapy were enrolled as the control group.

The Netrin-1 and UNC5b expression were evaluated in MDMs obtained from a subgroup of 10 control subjects and 29 CAD patients. The results obtained in CAD patients has been associated with the plaque characteristics assessed by OCT evaluation.

The exclusion criteria from the study were the presence of the previous history of CAD, severe chronic heart failure and heart valve disease, acute and chronic infections, liver diseases, neoplasia, immunologic disorders, surgical procedures, or trauma during the last 3 months and use of anti-inflammatory, immunosuppressive drugs, and antioxidant supplements.

Blood samples were collected in all patients prior to coronary angiography, and in the control group, samples were taken on a scheduled visit at the enrollment. The study was conducted according to the guidelines of the Declaration of Helsinki and approved by the Ethics Committee of Centro Cardiologico Monzino IRCCS. Informed consent was obtained from all subjects involved in the study.

### 2.2. Netrin Plasma Level Evaluation

Peripheral blood sample (30 mL) was collected from all participants to the study into vacutainer tubes containing ethylenediaminetetraacetic acid (EDTA) disodium salt (9.3 mM).

Plasma was obtained after whole blood centrifugation at 1200× *g* for 15 min and stored at −80 °C until analysis. The levels of Netrin-1 were measured using Netrin-1 (NTN1) ELISA Kit (MyBioSurce, SIAL s.r.l., Rome, Italy), following manufactory instructions.

### 2.3. Monocyte-Derived Macrophages (MDMs) Culture

Mononuclear cells were isolated from whole blood by density gradient centrifugation using Ficoll-Paque Plus (GE Healthcare, Milan, Italy). Lympho-monocytes were seeded in 35 mm plates (Primaria Corning, Sacco s.r.l., Como, Italy) at a density of 2 × 10^6^/mL and incubated for 90 min at 37 °C (5% CO_2_). After this time, cells were washed twice with phosphate-buffered saline (PBS) (Lonza, Milan, Italy) to remove non-adherent cells, and the adherent monocytes were cultured for 7 days in Medium 199 (Lonza, Milan, Italy) supplemented with 2 mM L-glutamine (Lonza, Milan, Italy), 100 U/mL penicillin, 100 µg/mL streptomycin (Lonza, Milan, Italy), and containing 10% autologous serum, to obtain MDMs [11]. MDMs morphology was assessed using phase-contrast microscopy (Axiovert 200 M; Zeiss, Milan, Italy) at 40× magnification, and MDMs with length >70 µm and a width <30 µm were defined spindle, whereas MDMs with similar width and length and >30–40 µm, were classified as round. Cells whose dimension and/or morphology did not meet these requirements were considered undefined.

### 2.4. Immunofluorescence Staining and Quantitative Analysis

MDMs were fixed with paraformaldehyde (PFA) 4% for 15 min at room temperature (RT) and were incubated with a monoclonal rabbit anti-human Netrin-1 antibody (1:100) (Abcam, Prodotti Gianni SRL, Milan, Italy), or with a polyclonal rabbit anti-human UNC5b antibody (1:100) (Abcam, Milan, Italy) overnight at 4 °C. Detection was performed with Alexa Fluor 488 (1:200, 60 min at room temperature (RT)) (Life Technologies Italia, Monza, Italy). Hoechst 33,258 (Merck Life Science SRL, Milan, Italy) was used for nuclei staining (1:10,000, 10 min at RT). For negative control experiments, the primary antibody was omitted. 

Fluorescence quantification was performed as previously described [10]. Data are expressed as a log of mean ± SD of fluorescence intensity/µm^2^ (AFU) for each MDM morphotype, subtracted from the value obtained from the negative control. At least five fields of view (400× magnification) were captured for each cell culture.

### 2.5. OCT Image Acquisition and Analysis

OCT examination was performed as previously reported [10]. Briefly, OCT images were acquired by C7 System (LightLab Imaging Inc/St Jude Medical, Westford, MA, USA) connected to a catheter C7 Dragonfly (LightLab Imaging Inc/St Jude Medical, Westford, MA, USA). The images were analyzed by two independent investigators (Institute of Cardiology, Catholic University of the Sacred Heart, Policlinico Gemelli, Rome, Italy). Plaque features (calcified, fibrous, or lipid plaques), measurement of fibrous cap thickness, detection of rupture, presence of intracoronary thrombi, intra-plaque microchannels, and the presence of macrophage infiltration (MØI) in the lesions were assessed as previously described [12,13,14,15]. A quantitative evaluation of macrophage content was obtained by measuring the OCT-derived tissue property index normalized standard deviation (NSD) [14,16].

### 2.6. Statistical Analysis

Continuous variables were expressed as mean ± (SD) or median with interquartile range (IQR), if they followed a normal or non-normal distribution, while categorical variables were shown as absolute numbers and percentages. Unpaired *t*-test or Wilcoxon’s rank-sum test were used to compare continuous variables between healthy subjects and CAD patients while chi-square test or Fisher’s exact test were performed for analyses that involved categorical variables. Comparisons among the groups were performed using ANOVA test for normally distributed variables and Wilcoxon’s rank-sum test for not normally distributed variables; Bonferroni’s correction for multiple comparisons was applied. Correlations between variables were executed using the Pearson’s test or the Spearman’s rank test, as appropriate. Statistical analyses were carried out with the SAS statistical package v.9.4 (SAS Institute Inc., Cary, NC, USA). All tests were two-sided, and *p* values < 0.05 were considered statistically significant.

## 3. Results

### 3.1. Study Population

Demographic, clinical, and laboratory characteristics of study participants are shown in Table 1. Even if the control group differed from patients for age and gender, the levels of Netrin-1 are not significantly correlated with these variables (Spearman’s correlation: r = −0.081, *p* = 0.526; r = −0.170, *p* = 0.181, respectively).

### 3.2. Netrin Plasma Levels

**Figure 1:**
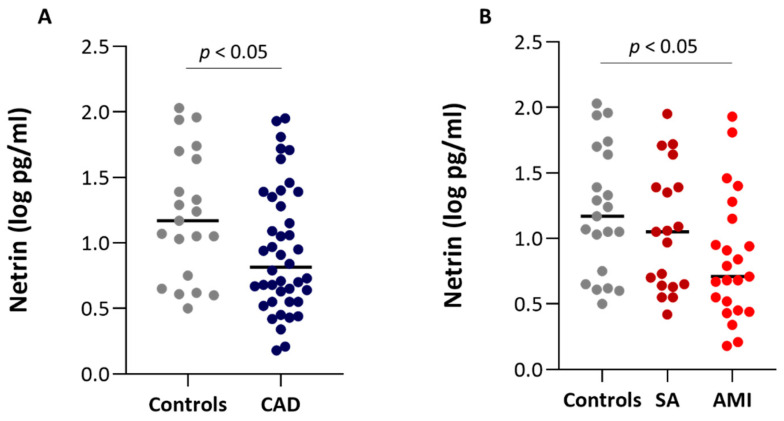
Plasma levels of Netrin-1 in the study population. (**A**) Plasma levels of Netrin-1 in control subjects (Controls; *n* = 21) and coronary artery disease (CAD) patients (*n* = 42). (**B**) Plasma levels of Netrin-1 in control subjects (n = 21), stable angina (SA) (*n* = 19) and acute myocardial infarction (AMI) (*n* = 23) patients. Comparisons were performed using Wilcoxon’s rank-sum test after Bonferroni’s correction.

Control subjects showed higher plasma levels of Netrin-1 as compared to CAD patients (Figure 1A) and a negative trend (r = −0.306, *p* = 0.014) is observed from controls to SA and AMI patients. In detail, a significant difference is observed in Netrin-1 plasma levels between control subjects and AMI patients but not between controls and SA patients (Figure 1B). The plasma levels of Netrin-1 were not different between SA and AMI patients. Interestingly, a negative correlation between levels of Netrin-1 and cardiovascular risk factors such as BMI and glycaemia were found (r = −0.437, *p* = 0.0003 and r = −0.316, *p* = 0.017, respectively). Moreover, circulating Netrin-1 was inversely associated with the presence of dyslipidemia (*p* < 0.05). Finally, although AMI patients showed higher levels of hs-CRP in comparison with SA patients, no significant difference in the levels of Netrin-1 was found in relation to the presence (C-reactive protein (CRP) > 2 mg/L) or absence (CRP < 2 mg/L) of systemic inflammation (0.94 ± 0.46 log pg/mL and 0.94 ± 0.54 log pg/mL, respectively).

### 3.3. Netrin-1 Expression in MDMs

**Figure 2:**
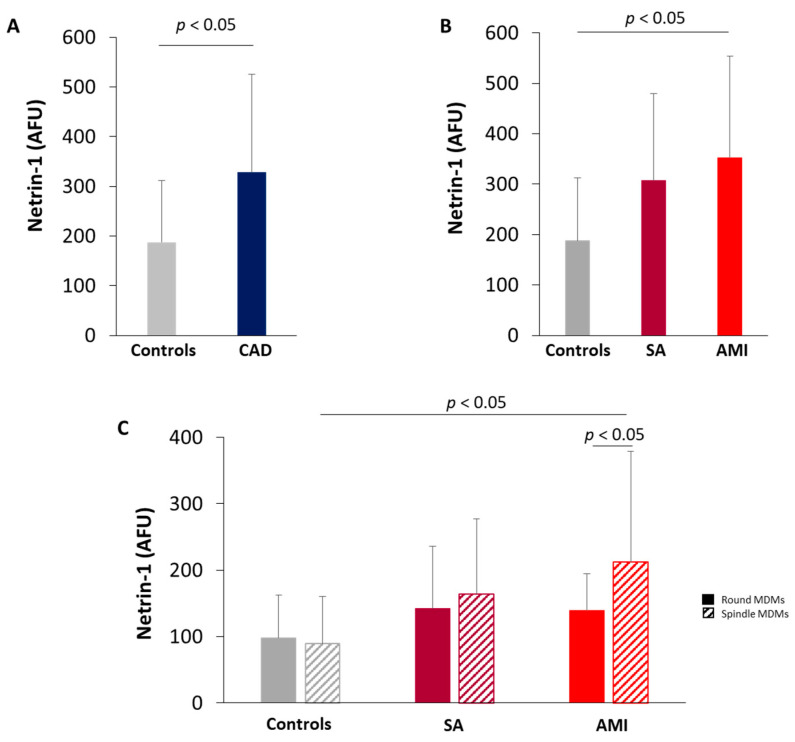
Quantitative analysis of Netrin-1. Netrin-1 levels in monocyte-derived macrophages (MDMs) obtained from (**A**) control subjects and CAD patients (Controls = 10; CAD = 29); (**B**) control subjects, SA, and AMI patients (Controls = 10; SA = 10; AMI = 19). (**C**) Netrin-1 levels in round and spindle MDM morphotypes in control subjects, SA, and AMI patients. Data are expressed as mean ± SD of fluorescence intensity/µm^2^ (AFU). At least three fields, 400× magnification, were analyzed.

MDMs obtained from monocytes isolated from CAD patients showed higher levels of Netrin-1 as compared with those from controls (Figure 2A). When analyzing CAD subgroups, a progressive increase in Netrin-1 levels was evidenced going from controls to SA and AMI patients (Figure 2B). The levels of Netrin-1 protein detected in the spindle and round MDM morphotypes were similar in all groups of subjects analyzed, but spindle MDMs of AMI patients showed a marked increase compared to spindle MDMs of control subjects (Figure 2C).

### 3.4. UNC5b Expression in MDMs

**Figure 3:**
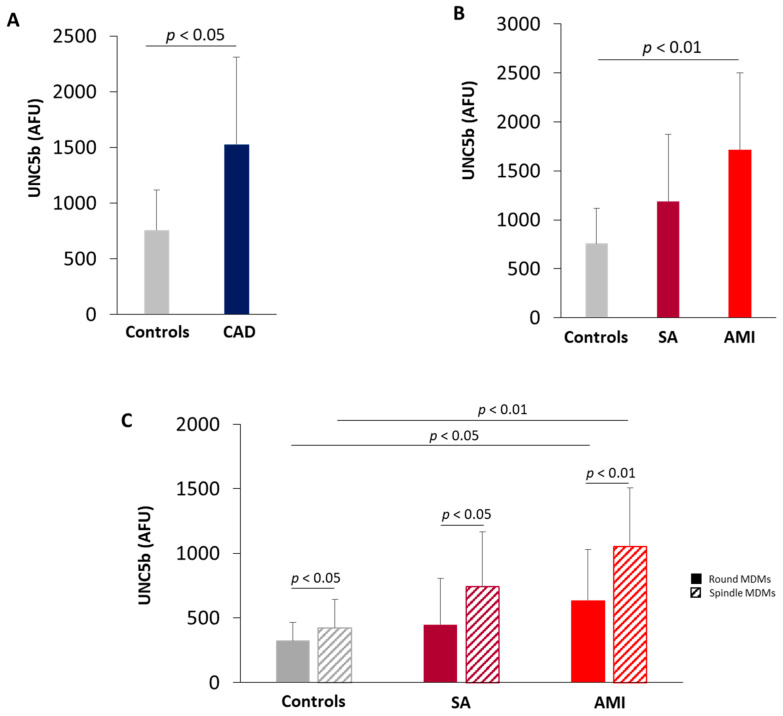
Quantitative analysis of UNC5b receptor. UNC5b levels in MDMs obtained from (**A**) Control subjects and CAD patients (Controls = 9; CAD = 28); (**B**) control subjects, SA, and AMI patients (Controls = 9; SA = 10; AMI = 18); (**C**) UNC5b levels in round and spindle MDMs obtained from control, SA and AMI patients. Data are expressed as mean ± SD of AFU (fluorescence intensity/µm^2^). At least three fields, 400× magnification, were analyzed.

MDMs obtained from CAD patients showed higher levels of UNC5b receptor as compared with MDMs of controls (Figure 3A). In detail, a progressive increase in UNC5b expression was detected going from control subjects to SA and AMI patients and the highest levels were evidenced in MDMs of AMI patients (Figure 3B). The receptor UNC5b is expressed both in spindle and round MDMs, but its levels were always higher in spindle MDMs compared to round MDMs. In addition, a significant increase in both spindle and round MDMs of AMI patients compared to control subjects has been detected (Figure 3C).

### 3.5. Correlation between the Intracellular Levels of Netrin-1 and Plaque Features Assessed by OCT

The coronary plaque features of CAD patients detected by OCT are shown in Table 2. The characteristics of the atherosclerotic plaque are similar between the two groups of patients except for the ruptured plaque and thrombus, which, as expected, are present in AMI patients.

**Figure 4:**
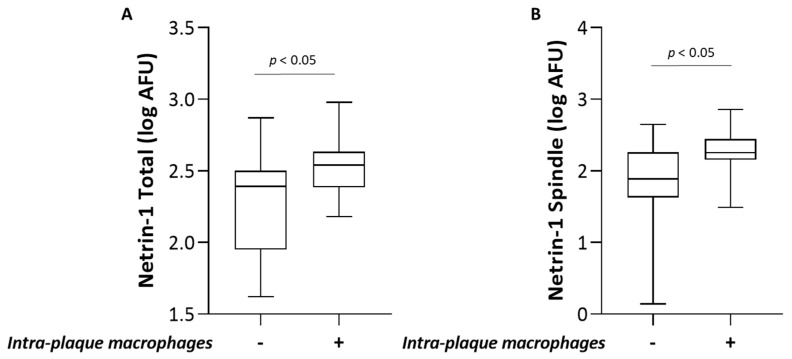
Association between intracellular Netrin-1 levels in MDMs and plaque feature analyzed by OCT. Association between intra-plaque macrophage infiltration and (**A**) Netrin-1 levels in MDMs and (**B**) spindle MDMs.

Interestingly, the macrophage infiltration in the coronary plaque was positively associated with the intracellular levels of Netrin-1 in MDMs (Figure 4A), particularly patients with macrophage infiltration in coronary plaque showed higher levels of Netrin-1 in MDMs compared to patients without macrophage infiltration. Moreover, Netrin-1 in spindle MDMs were positively associated with the macrophage accumulation (Figure 4B).

## 4. Discussion

In this study, we show that patients with CAD have lower levels of plasma Netrin-1 compared to control subjects, and in particular, the lowest levels have been detected in AMI patients. Moreover, a negative correlation between plasma levels of Netrin-1 and cardiovascular risk factors, such as BMI and glycemia, was highlighted. Accordingly, it has been reported that obese subjects had lower levels of circulating Netrin-1 compared to thin subjects [17]. Less clear is the information about the existence of a relationship between circulating Netrin-1 and glycemia. Therefore, our results are in contrast with the data reported by Yim et al. evidencing high serum Netrin-1 levels in subjects with impaired fasting glucose and with type 2 diabetes compare to normal controls [18], but they are in line with the reduced levels of plasma Netrin-1 detected in diabetic patients compared to healthy subjects [19].

Despite Netrin-1 plasma levels seem to be inversely correlated to the grade of atherosclerosis, in accordance with what was recently evidenced by Bruikman et al., we show that they do not allow to distinguish between SA and AMI patients [20]. Furthermore, although AMI patients have higher levels of hs-CRP compared to SA patients, no differences between the levels of circulating Netrin-1 in patients with evidence or not of systemic inflammation were found.

Several studies have shown that Netrin-1 released in the circulation by endothelial cells explicates anti-inflammatory and atheroprotective functions. Indeed, Netrin-1 stimulates NO production [21] and inhibits the adhesion of leukocytes, reducing their migration and infiltration into the vessel wall [4,5]. Monocyte migration and recruitment into the arterial intima is a crucial step in atherosclerotic plaque formation and progression, and studies in an experimental animal model have shown that the infusion of Netrin-1 induces protection against neointimal restenosis following endothelial damage [22]. Moreover, it has been evidenced that the overexpression of human Netrin-1 obtained in LDLR-/- mice via adenovirus delivery, reduced the monocyte/macrophages accumulation in the arteries [23]. In contrast, subjects with a genetic mutation of Netrin-1 characterized by a reduced capacity to bind the UNC5b receptor, showed an increase in monocyte adhesion and a reduction in macrophage migration, resulting in premature atherosclerosis [24]. On these bases, the lower plasma levels of Netrin-1 that we have detected in CAD patients could promote the monocyte migration to the inflammatory site, sustaining the progression of atherosclerotic plaque.

Plasma Netrin-1 is mainly produced by the endothelium; also macrophages express and secrete Netrin-1 and high levels have been detected in macrophage foam cells of human and mouse atherosclerotic plaque [6]. In addition, macrophages of plaque also express high levels of UNC5b receptor [8], the only Netrin-1 receptor express by leukocytes and responsible for the inhibitory effect of the Nertrin-1 on cell migration [2,25]. In this scenario, the Netrin-1/UNC5b signaling contributes to the disease progression by preventing macrophage egression. Interestingly, in our in vitro study, we detected high levels of both Netrin-1 and UNC5b in MDMs obtained from CAD patients compared to control subjects. Moreover, the levels of intracellular Netrin-1 in MDMs positively correlated with macrophage infiltration in coronary plaque detected by OCT analysis. Consistent with this, it is evidenced that the lack of Netrin-1 expression in macrophages of LDLR-/- mice promotes the emigration of macrophages from plaque resulting in a reduction of the size [6].

We have previously reported that spindle morphotypes of MDMs obtained from healthy subjects and from CAD patients showed a proinflammatory and proatherogenic profile compared to round, lipid-rich morphotypes [10,11]. Accordingly, also in this study, the proatherogenic characteristics of spindle MDMs are evidenced by higher levels of both Netrin-1 and UNC5b receptors. Moreover, a positive association between Netrin-1 levels in spindle MDMs and in vivo macrophage infiltration was showed.

In line with our results, recently it has been evidenced that foamy macrophages present in murine atherosclerotic plaque show a reduced expression of inflammatory genes compared to non-foamy macrophages that are characterized by higher levels of genes involved in the inflammatory process, including pro-inflammatory cytokine production, NF-κB signaling, and leukocyte recruitment [26].

Of course, our study shows some limitations, including (i) the small sample size reduces the general applicability of our results; (ii) although our in vitro model is commonly accepted by the scientific community to study tissue macrophages, it is possible that the in vitro macrophage phenotypes does not exactly reproduce the phenotypes generated in atherosclerotic plaque. Indeed, the environmental stimuli may affect the process of monocyte differentiation and macrophage functions; and (iii) OCT image interpretations may be suboptimal in the presence of residual thrombus, and macrophage infiltration might hamper the visualization of the underlying plaque features, nevertheless, intra-observer and inter-observer agreement were high.

Collectively, our data add important information about the role of Netrin-1 in the progression of atherosclerosis and suggest that its local inhibition may contribute to the reduction of plaque growth. In addition, our results further support the usefulness of MDMs obtained in vitro as a good representative model of plaque macrophage.

## Figures and Tables

**Table 1:** Baseline clinical, laboratory, and angiographic characteristics of the subjects. Data are expressed as mean ± SD or median and interquartile range. * *p* < 0.05 vs. Controls; ǂ *p* < 0.05 vs. SA. SA: stable angina; CAD: coronary artery disease; LVEF: left ventricular ejection fraction; WBC: white blood cells; RBC: red blood cells; LDL: low-density lipoprotein; HDL: high-density lipoprotein; hs-CRP: high-sensitive C-reactive protein: TnI: troponin-I; CK-MB: creatine phosphokinase-MB; LAD: left anterior descending; LCX: left circumflex; RCA: right coronary artery; ASA: aspirin; ACE-inhibitors, angiotensin-converting enzyme inhibitors.

Variables	Controls (*n* = 21)	CAD (*n* = 42)	*p* Value Controls vs. CAD	CAD		
				SA (*n* = 19)	AMI (*n* = 23)	ANOVA *p* Value
*Demographics*						
Age (years)	44.24 ± 9.73	62.76 ± 11.03	0.0001	64.84 ± 8.64 *	61.04 ± 12.60 *	0.0001
Male sex, *n* (%)	8 (38.0)	35 (83.3)	0.0003	14 (73.7)	21 (91.3) *	0.0003
Body mass index (kg/m^2^)	24.50 ± 3.61	29.95 ± 4.3	0.0001	29.11.0 ± 3.6 *	30.65 ± 4.88 *	0.0001
*Clinical characteristics*						
Current smoking, *n* (%)	3 (14.3)	23 (54.8)	0.0026	10 (52.6)	13 (56.5) *	0.007
Diabetes mellitus, *n* (%)	0	18 (42.8)	0.0002	7 (36.8)	11 (47.8)	0.003
Dyslipidemia, *n* (%)	2 (9.5)	21 (50.0)	0.0019	10 (52.6) *	11 (47.8) *	0.004
Hypertension, *n* (%)	1 (4.8)	21 (50.0)	0.0003	10 (52.6) *	11 (47.8) *	0.0006
Family history of CAD, *n* (%)	3 (14.3)	21 (50.0)	0.0066	7 (36.8)	14 (60.9) *	0.006
LVEF (%)	-	53.5 (45; 57)	-	55 (45; 57)	49 (45; 57)	0.4611
*Laboratory data*						
WBC (×10^9^/L)	6.4 (5.4; 6.6)	8.3 (7.5;10)	0.0001	8.4 (7.6; 11) *	7.6 (7.1; 9.6) *	0.0001
RBC (×10^12^/L)	4.7 (4.3; 5.2)	4.5 (4.0; 5.2)	0.161	4.3 (3.9; 4.7)	4.7 (4; 5.2)	0.068
Neutrophil count (×10^9^/L)	3.61 (3.1; 4.23)	5.1 (4.1; 6.8)	0.0001	5 (4.2; 7.8) *	5.1 (4, 6.8) *	0.0001
Lymphocyte count (×10^9^/L)	1.81 ± 0.54	2.22 ± 0.97	0.034	2.51 ± 0.95*	1.98 ± 0.94	0.026
Eosinophil count (×10^9^/L)	0.12 (0.09; 0.19)	0.2 (0.1; 0.3)	0.048	0.3 (0.1; 0.3) *	0.2 (0.1; 0.3)	0.051
Monocyte count (×10^9^/L)	0.38 (0.31; 0.46)	0.6 (0.5; 0.8)	0.0001	0.6 (0.5; 0.7) *	0.6 (0.5; 0.9) *	0.0001
Platelets (×10^9^/L)	236 (215; 271)	212 (184;268)	0.07	200 (154; 269)	212 (192; 268)	0.185
hs-CRP (mg/L)	-	2.65 (1.8; 16.9)		2.1 (1; 2.2)	13.6 (6; 21) ǂ	0.0001
Creatinine (mg/dL)	0.96 ± 0.48	0.98 ± 0.37	0.85	0.93 ± 0.29	1.02 ± 0.42	0.755
Glycaemia (mg/dL)	88.5 (84;98)	134.5 (110; 171)	0.0001	121 (104;140) *	147 (121; 187) * ǂ	0.0001
Total cholesterol (mg/dL)	200.71 ± 23.83	199.55 ± 46	0.89	186.0 ± 39.15	210.74 ± 48.98	0.132
LDL (mg/dL)	130.43 ± 23.35	119.05 ± 40.16	0.161	106.0 ± 29.75	129.83 ± 44.87	0.045
HDL (mg/dL)	54 (43; 59)	45 (41; 58)	0.24	48 (43; 61)	43 (36; 48)	0.054
Triglycerides (mg/dL)	87 (63; 129)	169 (113; 190)	0.0004	119 (82; 178)	186 (147; 214) *	0.0003
Peak TnI (μg/dL)	-	0.3 (0; 24.9)		0 (0; 0)	6 (1.3; 34.6) ǂ	0.0001
Peak CK-MB (μg/dL)	-	5 (1.9; 35.7)		2 (1.5; 2.21)	28 (12.3; 156.7) ǂ	0.0001
*Angiographic data*						
Culprit or treated vessel						
LAD, *n* (%)	0	32 (76.2)		11 (57.9)	21 (91.3)	
LCX, *n* (%)	0	2 (4.8)		2 (10.5)	0 (0)	
RCA, *n* (%)	0	8 (19.0)		6 (31.6)	2 (8.7)	
Multivessel disease, *n* (%)	0	27 (64.2)		16 (84.2)	11 (47.8) ǂ	0.02
*Admission therapy*						
ASA, *n* (%)	0	15 (35.7)		8 (42.1)	7 (30.4)	0.002
Beta-Blockers, *n* (%)	0	14 (33.3)		9 (47.3)	5 (21.7)	0.0006
ACE-inhibitors, *n* (%)	0	15 (35.7)		11 (57.9)	4 (17.4)	0.0001
Statins, *n* (%)	0	15 (35.7)		9 (47.4)	6 (26.1)	0.0009

**Table 2:** Optical coherence tomography features of coronary artery disease patients according to the clinical presentation. Continuous variables are expressed as median and interquartile range and categorical variables are expressed as percentage. MLA, minimal lumen area; TCFA, thin-cap fibroatheroma; NSD, normalized standard deviation.

Variables	SA (*n* = 19)	AMI (*n* = 23)	*p* Value
Lipid plaque, *n* (%)	14 (73.6)	18 (78.2)	0. 73
Fibrous plaque, *n* (%)	3 (15.8)	2 (8.7)	0.64
Calcific plaque, *n* (%)	4 (21.1)	4 (17.4)	1.0
Plaque rupture, *n* (%)	7 (36.8%)	18 (78.3)	0.006
Plaque erosion, *n* (%)	8 (42.1)	11 (47.8)	0.71
MLA, mm^2^	1.9 (1.7–3.6)	1.7 (1.1–3.9)	0.37
TCFA, *n* (%)	2 (10.5)	12 (52.2)	0.007
Thrombus, *n* (%)	1 (5.3)	16 (69.6)	0.0001
Lipid quadrants, *n*	2 (2–3)	3 (2–3)	0.06
Lipid arc degree, °	1215 (87–265)	260 (172–280)	0.006
Presence of microchannels, *n* (%)	5 (26.3)	13 (56.5)	0.049
Macrophage infiltration detection, *n* (%)	10 (56.2)	14 (60.9)	0.59
Macrophage NSD	6.4 (5.29–6.49)	6.5 (5.92–7.62)	0.21

## Data Availability

The datasets used and/or analyzed during the current study are available from the corresponding author on reasonable request.

## References

[1] Randolph G.J. (2008). Emigration of monocyte-derived cells to lymph nodes during resolution of inflammation and its failure in atherosclerosis. Curr. Opin. Lipidol..

[2] Ly N.P., Komatsuzaki K., Fraser I.P., Tseng A.A., Prodhan P., Moore K.J., Kinane T.B. (2005). Netrin-1 inhibits leukocyte migration in vitro and in vivo. Proc. Natl. Acad. Sci. USA.

[3] Kennedy T.E., Serafini T., de la Torre J.R., Tessier-Lavigne M. (1994). Netrins are diffusible chemotropic factors for commissural axons in the embryonic spinal cord. Cell.

[4] Van Gils J.M., Ramkhelawon B., Fernandes L., Stewart M.C., Guo L., Seibert T., Menezes G.B., Cara D.C., Chow C., Kinane T.B. (2013). Endothelial expression of guidance cues in vessel wall homeostasis dysregulation under proatherosclerotic conditions. Arterioscler. Thromb. Vasc. Biol..

[5] Lin Z., Jin J., Bai W., Li J., Shan X. (2018). Netrin-1 prevents the attachment of monocytes to endothelial cells via an anti-inflammatory effect. Mol. Immunol..

[6] van Gils J.M., Derby M.C., Fernandes L.R., Ramkhelawon B., Ray T.D., Rayner K.J., Parathath S., Distel E., Feig J.L., Alvarez-Leite J.I. (2012). The neuroimmune guidance cue netrin-1 promotes atherosclerosis by inhibiting the emigration of macrophages from plaques. Nat. Immunol..

[7] Rubina K.A., Tkachuk V.A. (2015). Guidance Receptors in the Nervous and Cardiovascular Systems. Biochemistry. Biokhimiia.

[8] Ramkhelawon B., Yang Y., van Gils J.M., Hewing B., Rayner K.J., Parathath S., Guo L., Oldebeken S., Feig J.L., Fisher E.A. (2013). Hypoxia induces netrin-1 and Unc5b in atherosclerotic plaques: Mechanism for macrophage retention and survival. Arterioscler. Thromb. Vasc. Biol..

[9] Yang X., Zhang J., Chen L., Yuan Z., Qin X., Wu Q., Shen D., He H., Yu C. (2018). The role of UNC5b in ox-LDL inhibiting migration of RAW264.7 macrophages and the involvement of CCR7. Biochem. Biophys. Res. Commun..

[10] Eligini S., Cosentino N., Fiorelli S., Fabbiocchi F., Niccoli G., Refaat H., Camera M., Calligaris G., De Martini S., Bonomi A. (2019). Biological profile of monocyte-derived macrophages in coronary heart disease patients: Implications for plaque morphology. Sci. Rep..

[11] Eligini S., Crisci M., Bono E., Songia P., Tremoli E., Colombo G.I., Colli S. (2013). Human monocyte-derived macrophages spontaneously differentiated in vitro show distinct phenotypes. J. Cell. Physiol..

[12] Scalone G., Niccoli G., Refaat H., Vergallo R., Porto I., Leone A.M., Burzotta F., D’Amario D., Liuzzo G., Fracassi F. (2017). Not all plaque ruptures are born equal: An optical coherence tomography study. Eur. Heart J. Cardiovasc. Imaging.

[13] Prati F., Regar E., Mintz G.S., Arbustini E., Di Mario C., Jang I.K., Akasaka T., Costa M., Guagliumi G., Grube E. (2010). Expert review document on methodology, terminology, and clinical applications of optical coherence tomography: Physical principles, methodology of image acquisition, and clinical application for assessment of coronary arteries and atherosclerosis. Eur. Heart J..

[14] Tearney G.J., Yabushita H., Houser S.L., Aretz H.T., Jang I.K., Schlendorf K.H., Kauffman C.R., Shishkov M., Halpern E.F., Bouma B.E. (2003). Quantification of macrophage content in atherosclerotic plaques by optical coherence tomography. Circulation.

[15] Tearney G.J., Regar E., Akasaka T., Adriaenssens T., Barlis P., Bezerra H.G., Bouma B., Bruining N., Cho J.M., Chowdhary S. (2012). Consensus standards for acquisition, measurement, and reporting of intravascular optical coherence tomography studies: A report from the International Working Group for Intravascular Optical Coherence Tomography Standardization and Validation. J. Am. Coll. Cardiol..

[16] Di Vito L., Agozzino M., Marco V., Ricciardi A., Concardi M., Romagnoli E., Gatto L., Calogero G., Tavazzi L., Arbustini E. (2015). Identification and quantification of macrophage presence in coronary atherosclerotic plaques by optical coherence tomography. Eur. Heart J. Cardiovasc. Imaging.

[17] Ramkhelawon B., Hennessy E.J., Menager M., Ray T.D., Sheedy F.J., Hutchison S., Wanschel A., Oldebeken S., Geoffrion M., Spiro W. (2014). Netrin-1 promotes adipose tissue macrophage retention and insulin resistance in obesity. Nat. Med..

[18] Yim J., Kim G., Lee B.W., Kang E.S., Cha B.S., Kim J.H., Cho J.W., Lee S.G., Lee Y.H. (2018). Relationship Between Circulating Netrin-1 Concentration, Impaired Fasting Glucose, and Newly Diagnosed Type 2 Diabetes. Front. Endocrinol..

[19] Liu C., Ke X., Wang Y., Feng X., Li Q., Zhang Y., Zhu J. (2016). The level of netrin-1 is decreased in newly diagnosed type 2 diabetes mellitus patients. BMC Endocr. Disord..

[20] Bruikman C.S., Vreeken D., Hoogeveen R.M., Bom M.J., Danad I., Pinto-Sietsma S.J., van Zonneveld A.J., Knaapen P., Hovingh G.K., Stroes E.S.G. (2020). Netrin-1 and the Grade of Atherosclerosis Are Inversely Correlated in Humans. Arterioscler. Thromb. Vasc. Biol..

[21] Zhang J., Cai H. (2010). Netrin-1 prevents ischemia/reperfusion-induced myocardial infarction via a DCC/ERK1/2/eNOS s1177/NO/DCC feed-forward mechanism. J. Mol. Cell. Cardiol..

[22] Liu N.M., Siu K.L., Youn J.Y., Cai H. (2017). Attenuation of neointimal formation with netrin-1 and netrin-1 preconditioned endothelial progenitor cells. J. Mol. Med. (Berl.).

[23] Khan J.A., Cao M., Kang B.Y., Liu Y., Mehta J.L., Hermonat P.L. (2011). Systemic human Netrin-1 gene delivery by adeno-associated virus type 8 alters leukocyte accumulation and atherogenesis in vivo. Gene Ther..

[24] Bruikman C.S., Vreeken D., Zhang H., van Gils M.J., Peter J., van Zonneveld A.J., Hovingh G.K., van Gils J.M. (2020). The identification and function of a Netrin-1 mutation in a pedigree with premature atherosclerosis. Atherosclerosis.

[25] Tadagavadi R.K., Wang W., Ramesh G. (2010). Netrin-1 regulates Th1/Th2/Th17 cytokine production and inflammation through UNC5B receptor and protects kidney against ischemia-reperfusion injury. J. Immunol..

[26] Kim K., Shim D., Lee J.S., Zaitsev K., Williams J.W., Kim K.W., Jang M.Y., Seok Jang H., Yun T.J., Lee S.H. (2018). Transcriptome Analysis Reveals Nonfoamy Rather Than Foamy Plaque Macrophages Are Proinflammatory in Atherosclerotic Murine Models. Circ. Res..

